# Monitoring of a micro-smart grid: Power consumption data of some machineries of an agro-industrial test site

**DOI:** 10.1016/j.dib.2016.12.033

**Published:** 2016-12-21

**Authors:** Enrico Fabrizio, Alessandro Biglia, Valeria Branciforti, Marco Filippi, Silvia Barbero, Giuseppe Tecco, Paolo Mollo, Andrea Molino

**Affiliations:** a Politecnico di Torino, Department of Energy (DENERG), Corso Duca degli Abruzzi 24, 10129 Torino, Italy; b University of Torino, Department of Agricultural, Forest and Food Sciences (DISAFA), L.go Paolo Braccini 2, Grugliasco, 10095 TO, Italy; c Agrindustria Tecco srl., Via Valle PO, 350 - Frazione Roata Rossi, 12020 Cuneo, Italy; d CSP – Innovazione nelle ICT, Via Nizza 150, Torino, Italy

## Abstract

For the management of a (micro)-smart grid it is important to know the patters of the load profiles and of the generators. In this article the power consumption data obtained through a monitoring activity developed on a micro-smart grid in an agro-industrial test-site are presented. In particular, this reports the synthesis of the monitoring results of 5 loads (5 industrial machineries for crop micronization, corncob crashing and other similar processes). How these data were used within a monitoring and managing scheme of a micro-smart grid can be found in (E. Fabrizio, V. Branciforti, A. Costantino, M. Filippi, S. Barbero, G. Tecco, P. Mollo, A. Molino, 2017) [1]. The data can be useful for other researchers in order to create benchmarks of energy use input appropriate energy demand values in optimization tools for the industrial sector.

**Specifications Table**

**Table:** 

Subject area	Engineering
More specific subject area	Energy engineering
Type of data	Graphs
How data was acquired	Survey, measurements [Wi-LEM (Wireless Local Energy Meters)]
Data format	Analyzed
Experimental factors	Data collected through power (3-phase high currents) monitoring
Experimental features	The system allows to measure continuously and in real-time the energy consumption of electricity loads through a network of wireless sensors that communicate using the IEEE 802.15.4 standard.
Data source location	Frazione Roata Rossi, Cuneo (Italy)
Data accessibility	Data is within this article

**Value of the data**

• Assessment of load profiles of some industrial machineries.
• May be used to create benchmarks of some industrial machineries.
• May be used as input of optimization models, such as [2], for industrial settlements.

## 1 Data

Data reported in this article refer to power consumption measurements. Different machineries were monitored in an agro-industrial test site and the data here presented allow to identify the pattern of load profile of the most important machines of the factory.

## 2 Experimental design, materials and methods

The power profiles were monitored through a Wi-LEM (Wireless Local Energy Meters) system, supplied by LEM, a worldwide leader in current transducers production. LEM current transducer are very useful in an industrial context where 3-phase high currents have to be measured, and they allow acquiring measurements without modifying the already existent plant wiring.

Data refer to the years 2013–2014 and were derived in order to characterize the power demand of the test-site. The monitoring was specifically put in place by the CSP partner. Considering the budget restrictions, the following machineries were monitored:

• Corncob crasher.
• Seeds micronizer at low temperature.
• Crasher F1.
• Crop micronizer.
• Crasher F2.

For each machinery, the design power and the measured one were compared. In particular, the following parameters were studied for each machinery:

• Peak power at the start up and duration.
• Mean power during normal working conditions and duration.
• Pattern of the load profile.
• Energy use.

In the following paragraphs, a summary of the results for each machinery is reported.

• Corncob crasher.

The total installed power to complete the various processing phases is equal to 216 kW. The mean monitored power during actual working conditions is around 90 kW. The peak power at the star-up is equal to 600 kW. The energy use, for a 10 h cycle is equal to 890 kWh.

**Fig. 1:**
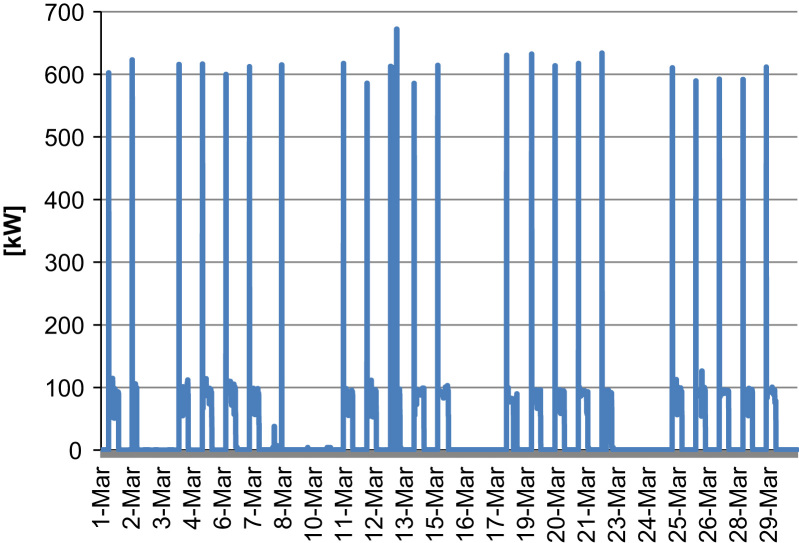
Power profile of the corncob crasher.

The profile pattern summarizes one month of measurements (March 2013) and it is very regular as can be seenin Fig. 1.

• Seeds micronizer at low temperature.

While the installed power of the machinery is equal to 90 kW the mean monitored power during actual working conditions was equal to 19 kW. The maximum monitored peak power was equal to 90 kW. The energy use can be estimated at 456 kWh/day.

**Fig. 2:**
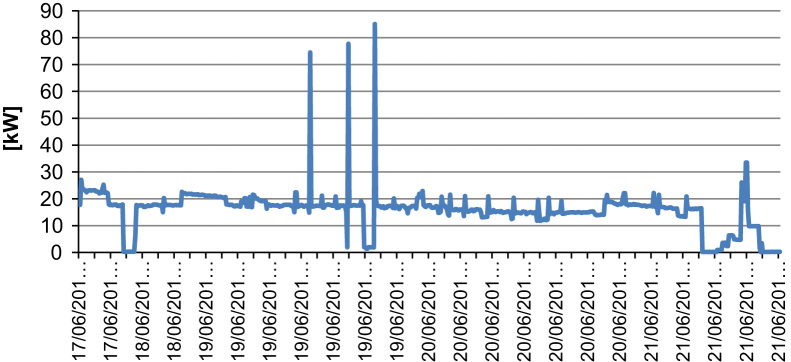
Power profile of low temperature seed micronizer.

The cycle is very irregular as can be seen from the graph of Fig. 2 (from 17 to 21 June 2013). No patterns can be identified. The machinery works continuously in different conditions.

• Crasher F1.

For this machinery total installed power and mean monitored power are very similar and equal to 25 kW. The peak power at the star-up is equal to 25 kW. The energy use can be estimated at 190 kWh/day.

**Fig. 3:**
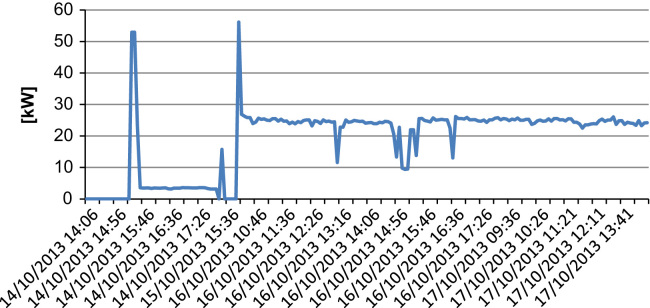
Power profile of the crasher F1.

The profile pattern irregular as can be seen from the graph of Fig. 3 that summarizes a period of measurement (14–17 October 2013)

• Crop micronizer.

The total installed power to complete the various processing phases is equal to 127 kW. The mean monitored power during actual working conditions is around 30 kW. The peak power at the star-up is equal to 90 kW. For this machinery, the available data were too scarce to identify energy use and typical load profiles.

• Crasher F2.

The total installed power to complete the various processing phases is equal to 45 kW. The machinery has two different working conditions (part load and full load). At part load, the mean monitored power during actual working conditions was equal to 35 kW with a spike peak power of 70 kW, while at full load the mean power is equal to 45 kW and the spike power was 100 kW. The mean daily energy use is equal to 840 kWh.

**Fig. 4:**
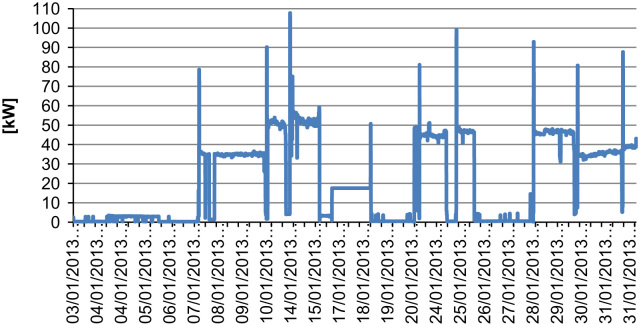
Power profile of the crasher F2.

The profile pattern is varied but some regularity can be found (e.g. two steps – full load and part load – of functioning) as can be seen from the graph of Fig. 4 that summarizes one month of measurements (January 2013).
