# Supplementary material for: Monitoring of a micro-smart grid: Power consumption data of some machineries of an agro-industrial test site
Source: Data Brief. 2016 Dec 21;10:564–8. doi: 10.1016/j.dib.2016.12.033 (PMC5219600; doi:10.1016/j.dib.2016.12.033)
Supplement: Supplementary file 1 — Supplementary material [file mmc1.docx]

Conflict of interests: none.

Enrico Fabrizio

8/12/2016
